# Influenza Vaccination Effectiveness in Paediatric ‘Healthy’ Patients: A Population-Based Study in Italy

**DOI:** 10.3390/vaccines10040582

**Published:** 2022-04-10

**Authors:** Anna Cantarutti, Elisa Barbieri, Fabio Didonè, Antonio Scamarcia, Carlo Giaquinto, Giovanni Corrao

**Affiliations:** 1 National Centre for Healthcare Research and Pharmacoepidemiology, Department of Statistics and Quantitative Methods, University of Milano-Bicocca, 20126 Milan, Italy; giovanni.corrao@unimib.it; 2 Unit of Biostatistics, Epidemiology and Public Health, Department of Statistics and Quantitative Methods, University of Milano-Bicocca, 20126 Milan, Italy; f.didone@campus.unimib.it; 3 Division of Pediatric Infectious Diseases, Department for Woman and Child Health, University of Padua, 35128 Padua, Italy; elisa.barbieri@unipd.it (E.B.); carlo.giaquinto@unipd.it (C.G.); 4 Società Servizi Telematici SRL—Pedianet, 35138 Padua, Italy; a.scamarcia@sosepe.com

**Keywords:** influenza vaccination, vaccine effectiveness, primary care, real-world evidence

## Abstract

Background: Seasonal influenza can cause serious morbidity, mortality, and financial burden in pediatric and adult populations. The influenza vaccine (IV) is considered the most effective way to prevent influenza and influenza-like-illness (ILI) complications. Objective: To assess the effectiveness of the IV in a cohort of healthy children in Italy. Methods: From the Pedianet database, all healthy children aged six months–14 years between 2009–2019 were enrolled. Cox proportional-hazards models were fitted to estimate hazard ratios and the 95% confidence interval for the association between IV exposure during each season of interest (from October to April of each year) with incident influenza/ILI. Exposure was considered as a time-varying variable. Vaccine effectiveness (VE) was calculated as (1-HR) × 100. The additive and prolonged effects of IV were evaluated across the seasons. Results: We found a high IV effectiveness among healthy children. No additional or prolonged effects were found. Conclusion: Our data indicates that IV was effective in preventing influenza/ILI in healthy children. Therefore, IV should be encouraged and provided free of charge to healthy children in all the Italian regions every year, reducing disease spread and lowering the burden on the pediatric population.

## 1. Introduction

Seasonal influenza can cause serious morbidity, mortality, and financial burden in pediatric and adult populations [1,2]. Before the COVID-19 pandemic, from 15 to 45% of children were infected yearly with influenza viruses and it has been estimated that most children have experienced infection at least once by six years of age [3]. In addition, viremic titers in children are higher than in adults, and the shedding of the virus is sustained for a longer period [4,5]. Therefore, children represent a critical source in influenza transmission and sustain annual epidemics [5].

Until 2018, 109.5 million influenza virus episodes were estimated globally among children under five years [6]. The incidence in high-income countries was 61.9 episodes per 1000 children per year (ranging from 32.5 to 117.9), with about 111 out of 1000 hospital admissions associated with influenza and with the highest incidence of hospitalized influenza episodes in children aged less than five months (4.4 (95% CI 3.1–6.3) per 1000 children per year). In Italy, in three consecutive seasons from 2013 to 2016, mortality rates ranged from 33–35 per 100,000 children per year in those younger than five years of age and were stable at four per 100,000 children per year in children 5 to 14 years of age [7]. 

The influenza vaccine (IV) is considered the most effective way to prevent influenza and influenza-related complications. Currently, in Italy, the IV mainly used is an inactivated influenza vaccine (IIV), administered intramuscularly, approved for children older than six months, with a live attenuated one (LAIV), administered intranasally, and approved for children older than two years. The IIV can contain three or four virus strains. The trivalent ones (IIV3) are currently targeted against the influenza A(H1N1) virus, the influenza A(H3N2) virus, and the influenza B lineage virus (B-Victoria or B-Yamagata), while the quadrivalent ones (IIV4), are targeted against both viruses A, and both viruses B. In Italy, the IIV4 has been available since the 2015–2016 flu season [8]. The influenza vaccine composition is updated annually by WHO based on global surveillance data [9]. The option of intranasal vaccination seemed to offer a more acceptable vaccination for children, as they are perceived to be less invasive [10,11]. Still, up to 2018, only five out of 30 EU/EEA member states (namely Finland, Austria, Latvia, Slovakia, Hungary, and the United Kingdom) offered seasonal IV to healthy children or adolescents free of charge. As a result, the IV coverage in the pediatric population is low [12]. This may also be due to a lack of data, awareness, or recognition of the burden of influenza in this age group. Childhood immunization against seasonal influenza promises to reduce the burden of disease through herd immunity; in Italy, annual seasonal IV is recommended to the entire population older than six months of age. Before 2019, the IV was offered free of charge at the point of delivery only to a part of the population with differences between regions (i.e., people older than 65 years of age, people with comorbidities, and all those at higher risk) [13,14]. 

Due to the high burden of influenza and the low IV coverage in the pediatric population, this study aims to assess the effectiveness of the influenza vaccine in preventing influenza infection in a cohort of healthy children six months to 14 years of age in Italy. We used the Pedianet database, a pediatric primary-care database collecting specific data from computerized clinical files of family pediatricians in Italy. 

## 2. Materials and Methods

### 2.1. Setting

In Italy, pediatric primary health care within the National Health System is provided free of charge by family pediatricians. This real-world observational study used data from the Pedianet database (http://www.pedianet.it, accessed on 1 March 2022). The Pedianet database encompasses an established primary care database with an organized network of >400 family pediatricians (FPs) across Italy who use the Junior Bit^®^ software in their clinical practice. Data generated by Pedianet FPs are anonymized, in compliance with Italian regulations, stored under a unique numerical identifier, and sent monthly to a centralized database in Padova for validation. The database includes patient demographics and clinical characteristics, including diagnoses (free text or coded using the 9th International Statistical Classification of Diseases and Related Health Problems system (ICD-9 CM) codes), drug prescriptions (coded by Anatomical Therapeutic Chemical (ATC) codes), healthcare co-payment exemptions, request to specialist visits, diagnostic procedures, hospital admissions, growth parameters, symptoms and/or other medical observations related to the visits, and the vaccination performed. Inclusion in the Pedianet database is voluntary; parents/legal guardians provided consent for their children’s anonymized data to be used for research purposes. Ethical approval of the study and the access to the database was approved by the Internal Scientific Committee of So.Se.Te. Srl, the legal owner of Pedianet.

### 2.2. Cohort Selection

This retrospective, observational study included children (i) followed by one of the FPs of Pedianet network adhering to the flu vaccination program (i.e., who actively vaccinated against influenza during the influenza season in agreement with the National Health System), (ii) who have been enrolled in Pedianet for at least one year and have at least two outpatient encounters, (iii) aged 6 months to 14 years of age during the observation period, that is between 1 October 2009, until 30 December 2019 (i.e., the influenza season goes from 1 October and 30 April of each year). All children with a chronic complex condition (e.g., cystic fibrosis, diabetes, chronic obstructive pulmonary disease, and asthma), immunodeficiency or immunosuppressive therapy, prematurity (less than 37 weeks’ gestation), Down syndrome, diabetes mellitus, renal failure, and congenital cardiac disease other than small ventricular septal defect were excluded from the analysis. 

### 2.3. Exposure to Influenza Vaccine

Exposed children were all children who received the influenza vaccine during at least one influenza season. The reference group consisted of children who had never received IV during the same period. 

### 2.4. Outcome 

The outcome of interest was to assess the effectiveness of the influenza vaccine in preventing the onset of influenza episodes and/or influenza-like illness (ILI) episodes. As repeated influenza episodes within the same season were rare (1.6%), we considered only one seasonal event for each patient [15]. 

An influenza episode was defined as any reported clinical diagnosis of influenza (ICD-9-CM code 487, 487.0, 487.1, 487.8) or free text of medical charts based on text strings containing the terms for flu (both in English and Italian such as “influenz” or “flu”). All results from the free-text search were individually evaluated for correct classification.

ILI was defined according to the European Centre for Disease Prevention and Control (ECDC) surveillance protocol and the InfluNet Protocol as the presence of (i) any free text of medical charts describing the sudden onset of symptoms, and (ii) at least one of the following terms describing systemic symptoms of fever or feverishness (i.e., temperature equal to or greater than 38 °C): malaise (i.e., general feeling of discomfort, illness, or unease whose exact cause is difficult to identify; tiredness, chills, myalgia/muscle weakness, headache; and (iii) at least one of the following terms describing respiratory symptoms: cough, sore throat, shortness of breath, or (iv) laryngitis, bronchitis, and or nausea and vomiting; and (v) the clinician’s judgment that the illness is due to an infection (i.e., a lower respiratory tract infection) [16,17].

### 2.5. Covariates 

Information on covariates used for confounding adjustment were obtained from the diagnoses and prescriptions registry. We considered demographic variables (age at the start of each influenza season, sex, region of birth), the number of primary care visits and the number of antibiotic therapies, influenza vaccine and influenza and/or influenza-like episodes in the season of interest, as well as in the season preceding the season of interest.

### 2.6. Statistical Analysis 

Descriptive statistics were provided for children exposed and unexposed to the influenza vaccine for each influenza season of interest (from 2009/2010 to 2018/2019). A chi-squared test and Student’s *t*-test were used for categorical covariates and continuous variables, respectively, to assess differences among exposed and unexposed children.

Cox proportional hazard models were separately fitted for each influenza season for estimating the hazard ratio (HR) and the corresponding 95% confidence interval (CI) of the association between the influenza vaccine and the onset of influenza and/or ILI. To avoid immortal time bias [18,19], the influenza vaccine exposure was considered as a time-varying covariate.

We used propensity score stratification (PSS) in the attempt at between-group balancing. First, we calculated the PS, namely the predicted probability of exposure to the influenza vaccine, through logistic regression models that included age at the start of each influenza season, sex, region of birth, number of primary care visits and antibiotic therapies recorded in the influenza season preceding the season of interest, and the presence of influenza/ILI episode and/or influenza vaccination in the season preceding the season of interest. Second, after omitting children in non-overlapping areas of the PS and ranking only the exposed children based on their PS, 10 equally sized PS strata were created and unexposed children were assigned to these strata based on their PS. Finally, weighted Cox regression models were used to derive an adjusted exposure effect after stratification, in which each exposed child received a weight of 1 and unexposed children were weighted in proportion to the distribution of the exposed in the stratum into which they fell [20]. Follow-up began at the beginning of each season and ended with death, migration, change to a pediatrician outside of the Pedianet network, incident influenza/ILI, or the end of follow-up (30 April). 

Vaccine effectiveness (VE) was calculated as (1-Hazard Ratio) × 100.

### 2.7. Sensitivity Analysis

**Figure 1:**
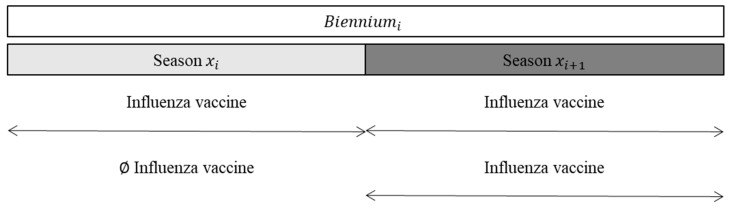
Definition of exposure to assess the additive effect of the influenza vaccine repeated in at least two consecutive seasons.

To assess (i) the additive effect of the influenza vaccination repeated in at least two consecutive seasons and (ii) the prolonged effect of the influenza vaccination received in the season preceding the season of interest on the following season, we performed two different analyses. For both analyses, we considered two-year seasons rather than a single season (the so-called biennium). For the first analysis, we selected children exposed to the influenza vaccine in the second year of each biennium, and we compared children exposed to the influenza vaccine in the first and second seasons of each biennium to those exposed to the influenza vaccine only in the second season (Figure 1).

**Figure 2:**
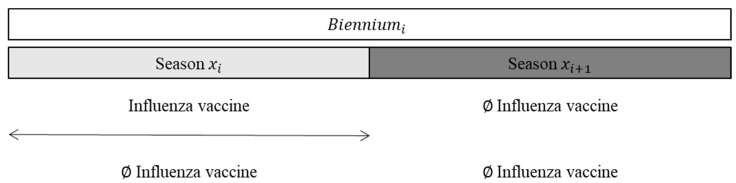
Definition of exposure to assess the prolonged effect of the influenza vaccine performed in a previous season on the following season.

For the second analysis, we selected children exposed to the influenza vaccine in the first season of each biennium, and we compared these children with those never exposed to the biennium (Figure 2).

Both the additional and the prolonged effect of the influenza vaccine were assessed through a Cox proportional hazard model considering the exposure as a fixed-time variable. The final model was adjusted by age and biennium.

All data analyses were performed using SAS statistical software, version 14.1 (SAS, College Station, TX, USA). Hypothesis tests were two-sided with a type I error of 0.05.

## 3. Results

**Figure 3:**
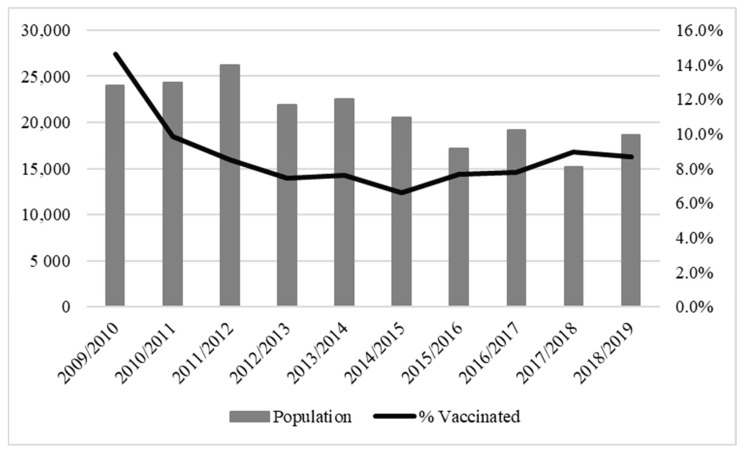
The trend of influenza vaccination among influenza seasons of interest from 1 October 2009 to 30 April 2019. Pedianet, 2009–2019.

**Figure 4:**
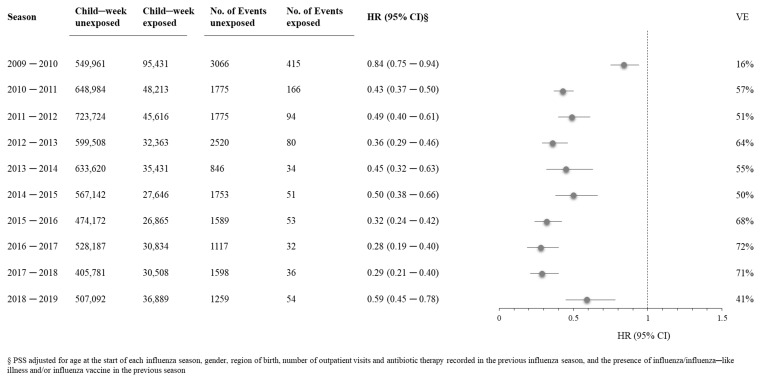
Adjusted hazard ratio and 95% CI for the association between influenza vaccination exposure during each season of interest (from October to April of each year) with influenza and influenza-related complications. Pedianet, 2009–2019.

Children included in our study ranged from 15,203 in the 2017–2018 season to 26,160 in the 2011–2012 season. The prevalence of influenza vaccinated children ranged from 15% in 2009–2010 to 7% in 2014–2015 (Figure 3). In general, vaccinated children were younger, more vaccinated for influenza and with fewer episodes of influenza/ILI in the preceding season, and with a greater number of antibiotic therapies and primary care visits (Supplementary Materials Table S1). The hazard ratios did not differ substantially according to unadjusted and adjusted estimates. Figure 4 shows the PSS adjusted hazard ratio (HR) for influenza/ILI outcomes and the vaccine effectiveness comparing children exposed to influenza vaccine with children unexposed. The higher VEs (>60%) were observed in the 2012–2013 (64%, 54% to 71%), 2015–2016 (68%, 58% to 76%), 2016–2017 (72%, 60% to 81%), and 2017–2018 (71%, 60% to 79%) seasons. The lowest VE was observed in the 2009–2010 season (16%, 6% to 25%). 

To assess the additive influenza vaccination effect, we restricted the cohort to those children exposed to influenza vaccine in the second season of each biennium considered (13,330) and we compared children exposed in both seasons of the biennium with those exposed only in the second season. Out of 10,202 (76%) exposed children, 3.8% recorded an episode of influenza/ILI in the second season of each biennium. Among unexposed children, 3128 recorded 129 (4.1%) episodes of influenza/ILI. Our results did not show any additive effect (HR: 0.91, 95% CI: 0.74 to 1.11) (Table 1). To evaluate the prolonged effect of the influenza vaccination, the cohort was restricted to those children exposed in the first season of each considered biennium. Out of 138,976 children 5138 were exposed to influenza vaccination in the first season of each biennium. Among them, 524 (10%) had influenza/ILI in the second season of each biennium. These children were compared with those who were never exposed during the biennium (133,838). Among them, 11,953 (9%) recorded an episode of influenza/ILI. We did not find any prolonged effect of influenza vaccination (0.97, 0.89 to 1.06) (Table 1).

## 4. Discussion 

Our findings showed a high influenza vaccine effectiveness among healthy children. Moreover, our results did not show additional or prolonged effects of the influenza vaccine, suggesting the importance of seasonal flu vaccination every year. 

Our study confirms and extends the findings of prior investigations that examined the clinical effectiveness of the influenza vaccine in children aged 0 to 14 years reporting contrasting results based on the patient population of interest, the number of vaccine doses, the influenza season, the method of ascertaining cases, and the definition of ILI. Estimates of effectiveness ranged from not effective (25%) to approximately 85% effective [21,22,23,24]. 

This variation in the effectiveness is mainly due to possible influenza strain circulation mismatch with strains contained in the IV and to other factors such as age class. 

According to yearly World Health Organization (WHO) recommendations for the northern hemisphere, a trivalent IV containing influenza A(H1N1)-like virus, influenza A(H3N2)-like virus, and influenza B-like virus was recommended for the season 2009–2010. However, in early 2009, the influenza A(H1N1)-pm09 virus began circulating, and by June 2009, the WHO declared a pandemic [25]. This virus had never been identified before and proved to be more transmissible and to cause a higher burden than influenza A(H1N1), especially in children and fragile populations [26]. Indeed, the circulating virus differed genetically from the vaccine strains, reducing VE. As a result, new IV, including the pandemic strain, were authorized in autumn 2009 in Europe [27]. Different from our findings, a systematic review assessing the influenza A(H1N1)-pm09 strain VE in the 2009–2010 season found rates varying from 48% to 89% in children and teenagers. However, a direct comparison is challenging because of different pandemic waves reported in the various countries, as well as the new vaccine’s late marketing in Italy that resulted in low coverage during the peak of the pandemic (the new vaccine was distributed starting from 15 October 2009 and the peak was observed in the week starting on 9 November 2009) [28]. 

In the 2014–2015 season, the ECDC reported that subtype A(H3N2) viruses were dominant in almost all reporting European countries, but that the majority of genetically characterised A(H3N2) viruses belonged to subgroups distinct from the currently recommended WHO vaccine-strain A/Texas/50/2012. In line with our finding reporting reduced VE in the season considered, a previous test-negative case control study conducted in Italy concluded that, even if the VE against A(H1N1)pdm09 and B viruses was good in the 2014–2015 season, the VE was low in Italy due to the antigenic and genetic mismatch between circulating A(H3N2) and the respective 2014/15 vaccine strain [29].

Finally, for the 2018–2019 season, a study conducted in six European countries concluded that the circulation of a newly emerged subclade for influenza A(H3N2) also reduced the VE among children aged 0–14 years (VE: 46%; 95% CI: 8–68), which is in line with our findings [30,31]. However, recently Koutskos et al. reported that the SARS-CoV-2 pandemic brought a notable global reduction in influenza cases and that the B/Yamagata lineage had not been isolated from April 2020 to August 2021, suggesting that this influenza lineage may have become extinct. This would provide opportunities for improving the availability and effectiveness of influenza vaccines [32].

With regard to the variation in VE in different age classes, a study conducted in Japan assessing IIV4 effectiveness in the 2018–2019 season reported higher effectiveness rates in younger children aged one to five years old compared to older children [33]. However, the VE variation based on age group may not be significant when considering the number of IV doses administered in the season. Indeed, according to a test-negative case-control study conducted in the USA during the 2014–2015 through 2017–2018 influenza seasons, younger IV naïve children seem to experience a lower effectiveness rate when considering a single-dose immunization cycle than older naive IV children [34].

A strength of our study was the use of the Pedianet primary-care database, which allowed us to study a very large population-based cohort, including sociodemographic characteristics, clinical information, and medicine utilization, to evaluate the effectiveness of IV among healthy children. Second, since children included in the study cohort were enrolled with family pediatricians (FPs) who adhered to the regional vaccination campaign, program data on vaccinations performed are of high quality. Indeed, FPs receive reimbursement for every vaccine administered with mandatory reporting on the number of doses administered. This allowed for the reduction in the misclassification of the exposure. Third, our study overcame the limitations of surveillance studies based on prospective data collection, reducing the underdetection of ILI cases. Moreover, we applied an algorithm on the clinical note-free text based on the ECDC ILI definition to reduce underdetection.

Our study also had several limitations. First, the study is of a retrospective nature which, as with any observational study, does not allow for the elimination of the possibility that patients receiving influenza vaccination differ from those who did not receive it for some unmeasured features that the pediatrician did not report in the medical records. Second, the exclusion of children of FPs who did not adhere to the regional vaccination campaign could have affected the study cohort. Third, because the outcome was based on the clinical assessment of the FPs rather than laboratory-confirmed influenza, the estimates of VE may vary based on a subjective evaluation of FPs. Finally, confounder variables were based on outpatient information. However, residual confounding might be present.

## 5. Conclusions

Despite these limitations, our data on influenza vaccination represents real-world evidence in the pediatric population, indicating that the IV was effective in preventing influenza/ILI in healthy children. Therefore, following the clinical evidence, influenza vaccination should be encouraged and provided free of charge to healthy children in all the Italian regions every year, thereby reducing the disease spread and lowering the burden in the pediatric population. 

## Figures and Tables

**Table 1:** Adjusted hazard ratio and 95% CI for evaluation of additive and prolonged effect of influenza vaccination among concomitant influenza seasons. Pedianet, 2009–2019.

	Children		Influenza and ILI			
	Exposed	Unexposed	Exposed	Unexposed	HR	(95% CI)
Additive effect	10,202	3128	388	129	0.91	(0.74–1.11)
Prolonged effect	5138	133,838	524	11,953	0.97	(0.89–1.06)

## Data Availability

The data used in this study cannot be made available in the manuscript, the supplemental files or in a public repository due to Italian data protection laws. The anonymized datasets generated during and/or analyzed during the current study can be provided on reasonable request, from the corresponding author, after written approval by the Internal Scientific Committee (info@pedianet.it).

## Supplementary Materials

The following supporting information can be downloaded at: https://www.mdpi.com/article/10.3390/vaccines10040582/s1, Table S1: Socio-demographic and clinical characteristics of the cohorts. Pedianet 2009–2019.
