# Supplementary material for: Influenza Vaccination Effectiveness in Paediatric ‘Healthy’ Patients: A Population-Based Study in Italy
Source: Vaccines (Basel). 2022 Apr 10;10(4):582. doi: 10.3390/vaccines10040582 (PMC9031219; doi:10.3390/vaccines10040582)
Supplement: Supplementary file 1 [file vaccines-10-00582-s001.zip › vaccines-1650745-supplementary.pdf]

Table S1. Socio-demographic and clinical characteristics of the cohorts. Pedianet 2009-2019.

	2009-2010 season					2010-2011 season				
	Unvaccinated		Vaccinated			Unvaccinated		Vaccinated		
	N=23,965				p-value	N=24,355				p-value
	20465 (85.4)		3500 (14.6)			21955 (90.15)		2400 (9.85)		
**Age in mo. - mean (std)**	80.12	(39.12)	71.24	(34.27)	<0.0001	80.32	(38.94)	74.06	(34.83)	<0.0001
**Follow-up in weks - mean (std)**	27.37	(7.63)	27.42	(7.53)	0.6869	28.55	(4.37)	29.32	(3.17)	<0.0001
**Gender**					0.0016					0.0084
female	9988	(48.81)	1607	(45.91)		10694	(48.71)	1101	(45.88)	
male	10477	(51.19)	1893	(54.09)		11261	(51.29)	1299	(54.13)	
**Influenza vaccine in the previous year**					<0.0001					<0.0001
No	19936	(97.42)	1868	(53.37)		20306	(92.49)	600	(25)	
Yes	529	(2.58)	1632	(46.63)		1649	(7.51)	1800	(75)	
**Influenza diagnosis in the previous year**					0.0142					0.9469
No	19054	(93.11)	3298	(94.23)		19358	(88.17)	2115	(88.13)	
Yes	1411	(6.89)	202	(5.77)		2597	(11.83)	285	(11.88)	
**No. of Antibiotic Therapy**					<0.0001					<0.0001
0	11774	(57.53)	1377	(39.34)		12102	(55.12)	1013	(42.21)	
1	4647	(22.71)	825	(23.57)		5151	(23.46)	551	(22.96)	
≥2	4044	(19.76)	1298	(37.09)		4702	(21.42)	836	(34.83)	
**No. of Antibiotic Therapy in the previous year**					<0.0001					<0.0001
0	11414	(55.77)	1243	(35.51)		11678	(53.19)	868	(36.17)	
1	4626	(22.6)	803	(22.94)		5132	(23.38)	547	(22.79)	
≥2	4425	(21.62)	1454	(41.54)		5145	(23.43)	985	(41.04)	
**No. of Outpatient Visits**					<0.0001					<0.0001
0	3782	(18.48)	16	(0.46)		3988	(18.16)	26	(1.08)	
1	3974	(19.42)	232	(6.63)		4290	(19.54)	203	(8.46)	
2	3360	(16.42)	444	(12.69)		3747	(17.07)	310	(12.92)	
≥3	9349	(45.68)	2808	(80.23)		9930	(45.23)	1861	(77.54)	
**No. of Outpatient Visits in the previous year**					<0.0001					<0.0001
0	3774	(18.44)	110	(3.14)		3616	(16.47)	55	(2.29)	
1	3711	(18.13)	292	(8.34)		3705	(16.88)	144	(6)	
2	3265	(15.95)	392	(11.2)		3420	(15.58)	237	(9.88)	
≥3	9715	(47.47)	2706	(77.31)		11214	(51.08)	1964	(81.83)	
**Place of Birth**					<0.0001					<0.0001
North	8013	(39.57)	1040	(29.86)		9221	(42.45)	847	(35.48)	
Island	1229	(6.07)	582	(16.71)		1122	(5.17)	448	(18.77)	
Center	4712	(23.27)	650	(18.66)		5085	(23.41)	311	(13.03)	
South	6295	(31.09)	1211	(34.77)		6295	(28.98)	781	(32.72)	
**Influenza and/or influenza-like ilness diagnosis**					0.7391					<0.0001
No	17992	(87.92)	3084	(88.11)		19161	(87.27)	2234	(93.08)	
Yes	2473	(12.08)	416	(11.89)		2794	(12.73)	166	(6.92)	

	2011-2012 season					2012-2013 season				
	Unvaccinated		Vaccinated			Unvaccinated		Vaccinated		
	N=26,160				p-value	N=21,928				p-value
	23940 (91.51)		2220 (8.49)			20300 (92.58)		1628 (7.42)		
**Age in mo. - mean (std)**	82.01	(39.21)	77.94	(35.86)	<0.0001	83.29	(39.48)	79.94	(36.39)	0.0009
**Follow-up in weks - mean (std)**	29.37	(3.38)	29.78	(2.63)	<0.0001	28.75	(3.81)	29.59	(2.6)	<0.0001
**Gender**					<0.0001					0.0401
female	11682	(48.8)	985	(44.37)		9826	(48.4)	745	(45.76)	
male	12258	(51.2)	1235	(55.63)		10474	(51.6)	883	(54.24)	
**Influenza vaccine in the previous year**					<0.0001					<0.0001
No	23190	(96.87)	566	(25.5)		19443	(95.78)	501	(30.77)	
Yes	750	(3.13)	1654	(74.5)		857	(4.22)	1127	(69.23)	
**Influenza diagnosis in the previous year**					<0.0001					<0.0001
No	20934	(87.44)	2054	(92.52)		18695	(92.09)	1556	(95.58)	
Yes	3006	(12.56)	166	(7.48)		1605	(7.91)	72	(4.42)	
**No. of Antibiotic Therapy**					<0.0001					<0.0001
0	14630	(61.11)	1047	(47.16)		12097	(59.59)	774	(47.54)	
1	5191	(21.68)	504	(22.7)		4585	(22.59)	380	(23.34)	
≥2	4119	(17.21)	669	(30.14)		3618	(17.82)	474	(29.12)	
**No. of Antibiotic Therapy in the previous year**					<0.0001					<0.0001
0	13017	(54.37)	886	(39.91)		12081	(59.51)	739	(45.39)	
1	5539	(23.14)	484	(21.8)		4378	(21.57)	366	(22.48)	
≥2	5384	(22.49)	850	(38.29)		3841	(18.92)	523	(32.13)	
**No. of Outpatient Visits**					<0.0001					<0.0001
0	4977	(20.79)	22	(0.99)		3956	(19.49)	24	(1.47)	
1	5048	(21.09)	222	(10)		4167	(20.53)	161	(9.89)	
2	4119	(17.21)	302	(13.6)		3514	(17.31)	252	(15.48)	
≥3	9796	(40.92)	1674	(75.41)		8663	(42.67)	1191	(73.16)	
**No. of Outpatient Visits in the previous year**					<0.0001					<0.0001
0	3850	(16.08)	56	(2.52)		3762	(18.53)	77	(4.73)	
1	4386	(18.32)	187	(8.42)		3891	(19.17)	151	(9.28)	
2	3919	(16.37)	262	(11.8)		3352	(16.51)	199	(12.22)	
≥3	11785	(49.23)	1715	(77.25)		9295	(45.79)	1201	(73.77)	
**Place of Birth**					<0.0001					<0.0001
North	10969	(46.3)	769	(34.84)		10096	(49.91)	731	(44.98)	
Island	1249	(5.27)	460	(20.84)		930	(4.6)	299	(18.4)	
Center	5045	(21.3)	256	(11.6)		3799	(18.78)	141	(8.68)	
South	6427	(27.13)	722	(32.71)		5404	(26.71)	454	(27.94)	
**Influenza and/or influenza-like ilness diagnosis**					<0.0001					<0.0001
No	22165	(92.59)	2126	(95.77)		17780	(87.59)	1548	(95.09)	
Yes	1775	(7.41)	94	(4.23)		2520	(12.41)	80	(4.91)	

	2013-2014 season					2014-2015 season				
	Unvaccinated		Vaccinated			Unvaccinated		Vaccinated		
	N=22,542				p-value	N=20,490				p-value
	20832 (92.41)		1710 (7.59)			19139 (93.41)		1351 (6.59)		
**Age in mo. - mean (std)**	85.51	(39.77)	81.72	(36.47)	0.0001	87.69	(39.92)	83.86	(36.98)	0.0006
**Follow-up in weks - mean (std)**	29.66	(2.47)	29.9	(1.78)	<0.0001	28.98	(3.82)	29.67	(2.53)	<0.0001
**Gender**					0.0508					0.6944
female	10038	(48.19)	782	(45.73)		9144	(47.78)	638	(47.22)	
male	10794	(51.81)	928	(54.27)		9995	(52.22)	713	(52.78)	
**Influenza vaccine in the previous year**					<0.0001					<0.0001
No	20401	(97.93)	545	(31.87)		18505	(96.69)	339	(25.09)	
Yes	431	(2.07)	1165	(68.13)		634	(3.31)	1012	(74.91)	
**Influenza diagnosis in the previous year**					<0.0001					0.0003
No	18329	(87.98)	1573	(91.99)		18311	(95.67)	1320	(97.71)	
Yes	2503	(12.02)	137	(8.01)		828	(4.33)	31	(2.29)	
**No. of Antibiotic Therapy**					<0.0001					<0.0001
0	13303	(63.86)	781	(45.67)		12243	(63.97)	686	(50.78)	
1	4333	(20.8)	363	(21.23)		3969	(20.74)	308	(22.8)	
≥2	3196	(15.34)	566	(33.1)		2927	(15.29)	357	(26.42)	
**No. of Antibiotic Therapy in the previous year**					<0.0001					<0.0001
0	12294	(59.01)	721	(42.16)		11637	(60.8)	568	(42.04)	
1	4738	(22.74)	404	(23.63)		4101	(21.43)	285	(21.1)	
≥2	3800	(18.24)	585	(34.21)		3401	(17.77)	498	(36.86)	
**No. of Outpatient Visits**					<0.0001					<0.0001
0	4360	(20.93)	14	(0.82)		3944	(20.61)	6	(0.44)	
1	4486	(21.53)	175	(10.23)		3969	(20.74)	145	(10.73)	
2	3628	(17.42)	253	(14.8)		3224	(16.85)	204	(15.1)	
≥3	8358	(40.12)	1268	(74.15)		8002	(41.81)	996	(73.72)	
**No. of Outpatient Visits in the previous year**					<0.0001					<0.0001
0	3821	(18.34)	63	(3.68)		3558	(18.59)	42	(3.11)	
1	4165	(19.99)	157	(9.18)		3768	(19.69)	132	(9.77)	
2	3552	(17.05)	238	(13.92)		3214	(16.79)	186	(13.77)	
≥3	9294	(44.61)	1252	(73.22)		8599	(44.93)	991	(73.35)	
**Place of Birth**					<0.0001					<0.0001
North	10893	(52.55)	699	(40.93)		8619	(45.21)	550	(40.77)	
Island	1557	(7.51)	392	(22.95)		1720	(9.02)	271	(20.09)	
Center	3738	(18.03)	99	(5.8)		4317	(22.64)	102	(7.56)	
South	4539	(21.9)	518	(30.33)		4408	(23.12)	426	(31.58)	
**Influenza and/or influenza-like ilness diagnosis**					<0.0001					<0.0001
No	19986	(95.94)	1676	(98.01)		17386	(90.84)	1300	(96.23)	
Yes	846	(4.06)	34	(1.99)		1753	(9.16)	51	(3.77)	

	2015-2106 season					2016-2017 season				
	**Unvaccinated**		**Vaccinated**			**Unvaccinated**		**Vaccinated**		
	**N=17,106**				p-value	**N=19,110**				p-value
	15796 (92.34)		1310 (7.66)			17627 (92.24)		1483 (7.76)		
**Age in mo. - mean (std)**	89.16	(40.1)	83.6	(37.75)	<0.0001	92.22	(39.87)	85.35	(38.01)	<0.0001
**Follow-up in weks - mean (std)**	29.24	(3.35)	29.87	(2.15)	<0.0001	29.21	(3.75)	29.82	(2.26)	<0.0001
**Gender**					0.1728					0.1774
female	7544	(47.76)	600	(45.8)		8344	(47.34)	675	(45.52)	
male	8252	(52.24)	710	(54.2)		9283	(52.66)	808	(54.48)	
**Influenza vaccine in the previous year**					<0.0001					<0.0001
No	15496	(98.1)	388	(29.62)		17367	(98.52)	516	(34.79)	
Yes	300	(1.9)	922	(70.38)		260	(1.48)	967	(65.21)	
**Influenza diagnosis in the previous year**					0.0005					<0.0001
No	14585	(92.33)	1244	(94.96)		15943	(90.45)	1391	(93.8)	
Yes	1211	(7.67)	66	(5.04)		1684	(9.55)	92	(6.2)	
**No. of Antibiotic Therapy**					<0.0001					<0.0001
0	10191	(64.52)	622	(47.48)		11975	(67.94)	719	(48.48)	
1	3286	(20.8)	308	(23.51)		3477	(19.73)	348	(23.47)	
≥2	2319	(14.68)	380	(29.01)		2175	(12.34)	416	(28.05)	
**No. of Antibiotic Therapy in the previous year**					<0.0001					<0.0001
0	9664	(61.18)	600	(45.8)		11250	(63.82)	667	(44.98)	
1	3423	(21.67)	291	(22.21)		3743	(21.23)	327	(22.05)	
≥2	2709	(17.15)	419	(31.98)		2634	(14.94)	489	(32.97)	
**No. of Outpatient Visits**					<0.0001					<0.0001
0	3263	(20.66)	9	(0.69)		3895	(22.1)	3	(0.2)	
1	3351	(21.21)	129	(9.85)		4151	(23.55)	142	(9.58)	
2	2711	(17.16)	178	(13.59)		3034	(17.21)	215	(14.5)	
≥3	6471	(40.97)	994	(75.88)		6547	(37.14)	1123	(75.72)	
**No. of Outpatient Visits in the previous year**					<0.0001					<0.0001
0	3002	(19)	40	(3.05)		3316	(18.81)	43	(2.9)	
1	3146	(19.92)	135	(10.31)		3605	(20.45)	140	(9.44)	
2	2541	(16.09)	183	(13.97)		3053	(17.32)	205	(13.82)	
≥3	7107	(44.99)	952	(72.67)		7653	(43.42)	1095	(73.84)	
**Place of Birth**					<0.0001					<0.0001
North	7168	(45.53)	490	(37.49)		8578	(48.81)	535	(36.1)	
Island	1052	(6.68)	306	(23.41)		1598	(9.09)	378	(25.51)	
Center	2719	(17.27)	60	(4.59)		3900	(22.19)	115	(7.76)	
South	4804	(30.52)	451	(34.51)		3497	(19.9)	454	(30.63)	
**Influenza and/or influenza-like ilness diagnosis**					<0.0001					<0.0001
No	14207	(89.94)	1257	(95.95)		16510	(93.66)	1451	(97.84)	
Yes	1589	(10.06)	53	(4.05)		1117	(6.34)	32	(2.16)	

	2017-2018 season					2018-2019 season				
	**Unvaccinated**		**Vaccinated**			**Unvaccinated**		**Vaccinated**		
	**N=15,203**				p-value	**N=18,589**				p-value
	13841 (91.04)		1362 (8.96)			16973 (91.31)		1616 (8.69)		
**Age in mo. - mean (std)**	94.4	(40.43)	86.48	(39.25)	<0.0001	94.54	(39.96)	84.67	(38.97)	<0.0001
**Follow-up in weks - mean (std)**	28.59	(4.41)	29.8	(2.11)	<0.0001	29.22	(3.39)	29.72	(2.33)	<0.0001
**Gender**					0.7508					0.3602
female	6556	(47.37)	639	(46.92)		8085	(47.63)	789	(48.82)	
male	7285	(52.63)	723	(53.08)		8888	(52.37)	827	(51.18)	
**Influenza vaccine in the previous year**					<0.0001					<0.0001
No	13533	(97.77)	318	(23.35)		16695	(98.36)	540	(33.42)	
Yes	308	(2.23)	1044	(76.65)		278	(1.64)	1076	(66.58)	
**Influenza diagnosis in the previous year**					<0.0001					<0.0001
No	12879	(93.05)	1306	(95.89)		15088	(88.89)	1505	(93.13)	
Yes	962	(6.95)	56	(4.11)		1885	(11.11)	111	(6.87)	
**No. of Antibiotic Therapy**					<0.0001					<0.0001
0	9422	(68.07)	708	(51.98)		12014	(70.78)	808	(50)	
1	2718	(19.64)	318	(23.35)		3088	(18.19)	355	(21.97)	
≥2	1701	(12.29)	336	(24.67)		1871	(11.02)	453	(28.03)	
**No. of Antibiotic Therapy in the previous year**					<0.0001					<0.0001
0	9007	(65.07)	616	(45.23)		11278	(66.45)	759	(46.97)	
1	2867	(20.71)	305	(22.39)		3511	(20.69)	378	(23.39)	
≥2	1967	(14.21)	441	(32.38)		2184	(12.87)	479	(29.64)	
**No. of Outpatient Visits**					<0.0001					<0.0001
0	2993	(21.62)	0	(0)		4028	(23.73)	0	(0)	
1	3371	(24.36)	153	(11.23)		4226	(24.9)	188	(11.63)	
2	2431	(17.56)	236	(17.33)		2956	(17.42)	224	(13.86)	
≥3	5046	(36.46)	973	(71.44)		5763	(33.95)	1204	(74.5)	
**No. of Outpatient Visits in the previous year**					<0.0001					<0.0001
0	2665	(19.25)	28	(2.06)		3361	(19.8)	54	(3.34)	
1	2984	(21.56)	123	(9.03)		3864	(22.77)	165	(10.21)	
2	2314	(16.72)	177	(13)		2989	(17.61)	233	(14.42)	
≥3	5878	(42.47)	1034	(75.92)		6759	(39.82)	1164	(72.03)	
**Place of Birth**					<0.0001					<0.0001
North	6484	(47.02)	461	(33.87)		8687	(51.6)	566	(35.18)	
Island	1074	(7.79)	362	(26.6)		1438	(8.54)	451	(28.03)	
Center	3064	(22.22)	94	(6.91)		2848	(16.92)	106	(6.59)	
South	3169	(22.98)	444	(32.62)		3863	(22.94)	486	(30.21)	
**Influenza and/or influenza-like ilness diagnosis**					<0.0001					<0.0001
No	12243	(88.45)	1326	(97.36)		15714	(92.58)	1562	(96.66)	
Yes	1598	(11.55)	36	(2.64)		1259	(7.42)	54	(3.34)	
